# Supplementary material for: The interactome of intact mitochondria by cross-linking mass spectrometry provides evidence for coexisting respiratory supercomplexes
Source: Mol Cell Proteomics. 2017 Dec 8;17(2):216–32. doi: 10.1074/mcp.RA117.000470 (PMC5795388; doi:10.1074/mcp.RA117.000470)
Supplement: Supplemental Data [file supp_RA117.000470_133922_0_supp_23978_fzffwf.zip › spectra_annotation/mito_DR_spectra_annotation/9-1-1-1-1-1.pdf]

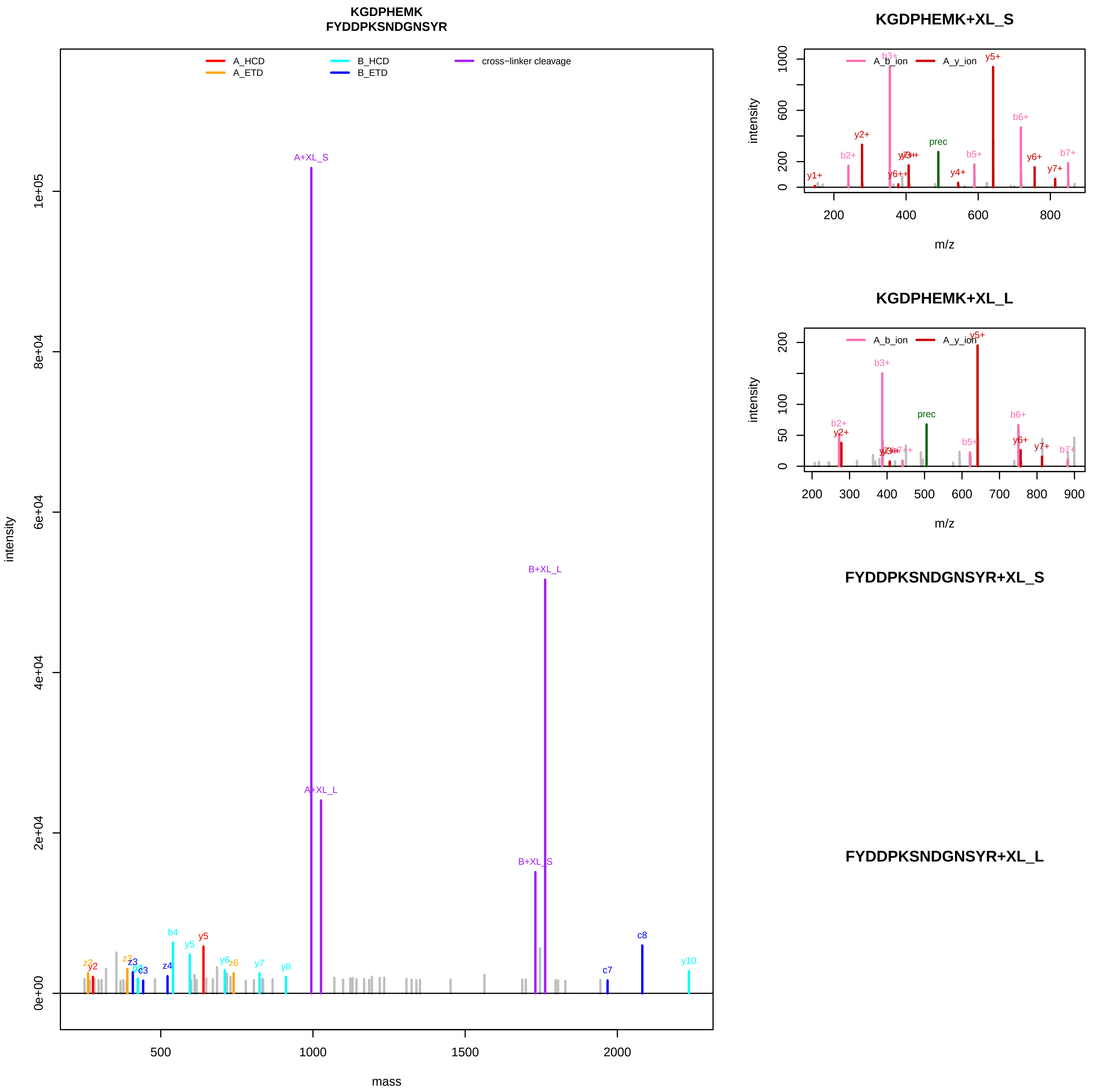
KGDPHEMK
FYDDPKSNDGNSYR
A_HCD
A_ETD
B_HCD
B_ETD
cross−linker cleavage
A+XL_S
B+XL_L
A+XL_L
B+XL_S
z2
y2
z3
z3
c3
z4
b4
y5
y5
y6
z6
y7
y8
c7
c8
y10
intensity
0e+00
2e+04
4e+04
6e+04
8e+04
1e+05
500
1000
1500
2000
mass
KGDPHEMK+XL_S
A_b_ion
A_y_ion
b3+
y5+
b6+
y2+
prec
y3+
b2+
b5+
y6+
b7+
y7+
y1+
y6++
y4+
intensity
0
200
600
1000
200
400
600
800
m/z
KGDPHEMK+XL_L
A_b_ion
A_y_ion
y5+
b3+
b2+
y2+
prec
b6+
b5+
y6+
y7+
b7+
intensity
0
50
100
200
200
300
400
500
600
700
800
900
m/z
FYDDPKSNDGNSYR+XL_S
FYDDPKSNDGNSYR+XL_L
